# Is Percutaneous Mechanical Atherothrombectomy a Durable, Cost-Effective Solution for the Treatment of Femoropopliteal In-Stent Reocclusion?

**DOI:** 10.1016/j.jscai.2025.103669

**Published:** 2025-06-17

**Authors:** Thomas Zeller

**Affiliations:** University Hospital Freiburg, Department Heart-Center Freiburg—Bad Krozingen, Bad Krozingen, Germany

In-stent restenosis (ISR), in particular, Tosaka class II (diffuse ISR) and class III (total occlusion ISR) lesions,^1^ represents the most challenging lesion population in endovascular treatment of the femoropopliteal artery due to limited durability of most interventional techniques. Plain old balloon angioplasty (POBA) with or without vessel preparation, such as laser angioplasty, resulted in 1-year primary patency of roughly 25% to 40%.^2^ Drug-eluting techniques such as paclitaxel drug-coated balloons (DCBs) or relining the ISR with a second paclitaxel-releasing drug-eluting stent have shown to improve short-term outcomes compared with POBA, with 1-year patency rates ranging from 70.5% to 88.7% for DCBs and 78.8% for drug-eluting stent.^3^, ^4^, ^5^ Additionally, relining the stent with an endoprosthesis has shown superior 2-year clinical outcomes compared with POBA.^6^

Debulking to remove the occlusive material from the stent lumen (consisting of neointima and thrombus) seems to be an attractive treatment tool to potentially improve the durability of endovascular revascularization of in-stent occlusions. In this issue of *JSCAI*, Stanberry et al^7^ calculated the costs of the treatment of femoropopliteal ISR using a mechanical rotational aspiration atherothrombectomy device (Rotarex S; BD) with or without adjunctive POBA or bare metal stenting and compared these costs with those of arterial bypass surgery. These costs were imputed in a United Kingdom–specific budget impact analysis comparing day case–based percutaneous mechanical atherothrombectomy (PMA) and inpatient surgical therapy of femoropopliteal ISR from the perspective of the vascular service (health care provider) over a 5-year time horizon. As a result, the analysis estimated that the introduction of PMA into future clinical UK practice at a typical vascular service could achieve a cost-savings of £4750 for each patient with femoropopliteal ISR undergoing an endovascular procedure instead of bypass surgery. This resulted in a total budget impact of £142,497 over the model’s 5-year time horizon. Reductions in postsurgical length of stay, delayed discharges, and operating theater utilization were the major drivers of cost-savings for the health care provider.

A few limitations should be considered. Calculating only the PMA-related device costs for the budget calculation is a UK-specific scenario. In other countries, additional devices such as percutaneous transluminal angioplasty balloons, DCB, and stents for day case procedures may not be fully covered by the individual health care system, and these costs may change the outcome of the budget impact analysis. As such, the study findings are very likely country specific and may need to be recalculated for other health care systems. Moreover, the Rotarex S device is available in 6F and 8F catheter sizes. Not every patient qualifies for a day case procedure, in particular, if an 8F sheath is needed. Finally, the health care providers’ cost perspective is of interest for budget negotiations between health care providers and payers parties; however, regarding the global cost burden, the payer’s perspective is more relevant considering the limited financial resources of health care systems globally. In particular, it should be noted that mechanical debulking of ISR lesions without using additional antiproliferative drug releasing devices has shown disappointing mid-term outcomes. A very first analysis of the performance of the early 8F catheter version of the Rotarex device with or without POBA published in 2002 resulted in inferior 1-year patency results, comparing ISR with native femoropopliteal lesions (26% vs 67%, respectively).^8^ Similarly, in the EXITE trial (excimer laser randomized controlled study for the treatment of femoropopliteal in-stent restenosis), excimer laser debulking plus POBA did not result in superior 6-month patency outcomes compared with POBA without vessel preparation; the patency difference of 71.1% vs 56.4%% was driven by a higher periprocedural treatment success of the laser cohort.^2^ These low patency results (1) mandate the additional use of drug-coated devices and (2) may result in a high rate of secondary, potentially open surgical, redo procedures following PMA, thereby increasing the global health care cost burden.
